# Bacteria-Specific Neutrophil Dysfunction Associated with Interferon-Stimulated Gene Expression in the Acute Respiratory Distress Syndrome

**DOI:** 10.1371/journal.pone.0021958

**Published:** 2011-07-06

**Authors:** Kenneth C. Malcolm, Jennifer E. Kret, Robert L. Young, Katie R. Poch, Silvia M. Caceres, Ivor S. Douglas, Chris D. Coldren, Ellen L. Burnham, Marc Moss, Jerry A. Nick

**Affiliations:** 1 Department of Medicine, National Jewish Health, Denver, Colorado, United States of America; 2 Division of Pulmonary Science and Critical Care Medicine, Department of Medicine, University of Colorado Denver School of Medicine, Aurora, Colorado, United States of America; 3 Pulmonary and Critical Care, Department of Medicine, Denver Health Medical Center, Denver, Colorado, United States of America; University of Giessen Lung Center, Germany

## Abstract

Acute respiratory distress syndrome (ARDS) is a poorly understood condition with greater than 30% mortality. Massive recruitment of neutrophils to the lung occurs in the initial stages of the ARDS. Significant variability in the severity and duration of ARDS-associated pulmonary inflammation could be linked to heterogeneity in the inflammatory capacity of neutrophils. Interferon-stimulated genes (ISGs) are a broad gene family induced by Type I interferons. While ISGs are central to anti-viral immunity, the potential exists for these genes to evoke extensive modification in cellular response in other clinical settings. In this prospective study, we sought to determine if ISG expression in circulating neutrophils from ARDS patients is associated with changes in neutrophil function. Circulating neutrophil RNA was isolated, and hierarchical clustering ranked patients' expression of three ISGs. Neutrophil response to pathogenic bacteria was compared between normal and high ISG-expressing neutrophils. High neutrophil ISG expression was found in 25 of 95 (26%) of ARDS patients and was associated with reduced migration toward interleukin-8, and altered responses to *Staphylococcus aureus*, but not *Pseudomonas aeruginosa*, which included decreased p38 MAP kinase phosphorylation, superoxide anion release, interleukin-8 release, and a shift from necrotic to apoptotic cell death. These alterations in response were reflected in a decreased capacity to kill *S. aureus*, but not *P. aeruginosa*. Therefore, the ISG expression signature is associated with an altered circulating neutrophil response phenotype in ARDS that may predispose a large subgroup of patients to increased risk of specific bacterial infections.

## Introduction

The acute respiratory distress syndrome (ARDS) affects over 150,000 people per year, and is characterized by diffuse pulmonary infiltrates on chest radiograph, severe hypoxia, and respiratory failure with a mortality of 30–40%. Diverse insults are capable of initiating ARDS including pneumonia, physical lung injury, sepsis, and trauma. ARDS is distinguished by variability in susceptibility, severity, and outcomes; however, mechanisms that account for this variability are unclear. Known contributors to heterogeneity in ARDS include demographic and clinical factors [1]–[3], and coding variation in genes within the inflammatory response and coagulation pathways [4], [5]. For several of these mechanisms, the potential predisposition towards developing ARDS is either present throughout the life of the patient, or is a chronic condition that would not be expected to resolve over time. However, ARDS rarely reoccurs within an individual suggesting that mechanisms also exist that sporadically place subjects at increased risk.

As massive accumulation of neutrophils to the lung is an early feature of ARDS [6], heterogeneity in the inflammatory capacity of the neutrophil could determine the extent, severity and duration of pulmonary inflammation. In many neutrophil-mediated diseases, the protective benefit of pathogen killing is balanced against the considerable capacity of the cell to inflict tissue injury [7], [8]. Dysregulation of neutrophil function in the inflammatory environment of ARDS is supported by reports of worse outcomes associated with elevated levels of neutrophil-related factors in the circulation or airways [9]–[13], and functional changes in neutrophils from ARDS patients [14]–[17]. Thus, much evidence suggests that both over-exuberant and/or diminished responses by the innate immune system can result in worse clinical outcomes in severely ill patients. However, it is still not possible to predict which patients will exhibit susceptibility to severe lung injury or neutrophil dysfunction. Increasingly, it has become evident that neutrophils, and other cells of the innate immune system, are capable of complex “adaptive” responses to pathogens by either decreasing (tolerance) or increasing (priming) their response to challenges [18], [19]. It now seems clear that neither priming nor tolerance occur in isolation, and a reprogramming of cellular phenotype is possible in response to various sequences of infectious challenges [18], [19].

Interferon-stimulated genes (ISGs) are upregulated in response to Type I interferons, IFNα and IFNβ [20], and include *MX1*, *ISG15* and *IFIT1* [21]. Viral factors, intracellular bacteria, and systemic autoimmune disorders evoke production of the IFNα/β and ISGs [20]. However, ISG expression in the blood and leukocytes serves as a well-validated marker of recent stimulation by IFN α/β as a result of viral, but not bacterial, infection *in vivo* [22]–[25]. Normally, ISG expression confers protection from ongoing and subsequent viral infection [20]; however, the well-recognized susceptibility to *Staphylococcus aureus* and other bacterial infections following influenza infection suggests that post-viral innate immunity is complex [26]. The possibility that ISG expression could transiently affect neutrophil response has received little attention, and the impact of ISG expression on neutrophil responses to bacteria is not known. Moreover, the role of ISG expression in ARDS patients has not been described.

We hypothesize that high ISG expression accompanies a transient reprogramming of the circulating neutrophil phenotype in the context of the inflammatory environment of ARDS, which results in an impaired ability to kill pathogenic bacteria. Herein, we show a subset of ARDS patients displayed elevated ISG expression. Circulating neutrophils from ARDS patients with high ISG expression demonstrated blunted activation of the p38 MAP kinase (MAPk) cascade in response to *S. aureus*, and a decrease in p38 MAPk-mediated responses. Of primary importance, high ISG expression in neutrophils from ARDS patients also had reduced killing of *S. aureus*, but not *Pseudomonas aeruginosa*. The neutrophil-mediated inflammatory response to bacteria associated with ISG expression has not been described previously, and highlights the potential for previous viral infection to selectively impact specific innate immune responses in ARDS.

## Materials and Methods

### Ethics Statement

The study protocol was approved by the Colorado Multiple Institutional Review Board (COMIRB) committee and the National Jewish Health Institutional Review Board; all subjects, or an appropriate proxy, gave written informed consent. The study was conducted in accordance with the Declaration of Helsinki.

### Subject enrollment and sample collection

Circulating neutrophils from ARDS patients were isolated from whole blood obtained within 72 h of patients enrollment into one of three NHLBI ARDS Network studies conducted at the University of Colorado affiliated hospitals, each with identical enrollment criteria. The parent studies were: Drug Study of Albuterol to Treat ALI (ALTA, ClinicalTrials.gov Identifier: NCT00434993), Early Versus Delayed Enteral Feeding and Omega-3 Fatty Acid/Antioxidant Supplementation for Treating People With ALI or ARDS (EDEN-Omega Study: NCT00609180), or Early Versus Delayed Enteral Feeding to Treat People With ALI or ARDS (The EDEN Study: NCT00883948). Sample processing and data analysis was performed at NJH, with approval by the National Jewish Health Institutional Review Board (HS-2342CO). This ancillary trial was also registered in ClinicalTrials.gov (Identifier: NCT00548795). The central inclusion criteria were that within a 24-hour time period patients demonstrate an acute onset of hypoxia (PaO_2_/FiO_2_≤271 – adjusted for Denver altitude of 1600 m), bilateral infiltrates by chest radiograph, and requirement for intubation and positive pressure ventilation in the absence of evidence for left-sided cardiac failure. All patients enrolled into the parent studies were eligible for this ancillary study, and selection was not based on history or clinical evidence of recent or ongoing viral infection. Diagnostic testing for acute or chronic viral infections was performed on a limited basis in the context of clinical care, and not as part of the study protocol.

### Isolation of neutrophils and RNA

Human neutrophils were isolated from peripheral blood using the plasma Percoll method [27]. RNA was extracted immediately from 10−20×10^6^ isolated neutrophils using TRIzol reagent [28].

### Definition of high ISG expression

Gene expression was quantified by real-time PCR of *MX1, IFIT1*, and *ISG15* relative to *GAPDH* by the ΔCt method using standard conditions. Primers and probes were obtained from Applied BioSystems (*MX1*, Hs00182073_m1; *ISG15*; Hs00192713_m1; *GAPDH* Endogenous Control) and Roche (*IFIT1*, Universal Probe Library #9 and forward: 5′-AGAACGGCTGCCTAATTTACA-3′; reverse: 5′-GCTCCAGACTATCCTTGACCT-3′). Expression of each ISG was normalized to GAPDH expression, followed by log_2_ transformation. Identification of the high ISG expression cohort was performed by hierarchical clustering using Multi Experiment Viewer [29], using Euclidean distance analysis and the complete linkage method.[29] To confirm assignment of samples, three-node *k*-means clustering was performed, also using Multi Experiment Viewer. The entire 25 subjects that were grouped by hierarchical clustering were confirmed to be present in a single node by *k*-means clustering.

### Bacterial cultures and killing

A clinical pulmonary isolate of a methicillin-sensitive *S. aureus* was grown to 10^8^ cfu/ml in tryptic soy broth, stored in aliquots of 15% glycerol in 0.9% saline and thawed immediately before use. *Pseudomonas aeruginosa* (strain PAO1) was grown in overnight culture in RPMI containing 2% heat-inactivated platelet-poor plasma (HIPPP) and adjusted to an OD_600_ of 0.30 (corresponding to 5×10^8^ cfu/ml) before dilution. Neutrophil killing of *S. aureus* and *P. aeruginosa* was determined by the growth of bacteria from wells with neutrophils compared to growth of control wells of bacteria without neutrophils. Briefly, 10^5^ neutrophils in 100 µl RPMI medium were allowed to settle for 30 min in duplicate wells of a 96-well plate. A bacteria-to-neutrophil ratio of 1∶1 was contact-synchronized by centrifugation at 110 *g* for 3 min. Control reaction contained no neutrophils. Bacteria/neutrophil co-cultures (or controls with only bacteria) were incubated at 37°C for 1 h. Killing reactions were stopped with 1% Triton X-100, and cultures were supplemented with 1X LB and 20 µl volume alamarBlue reagent[30] in a final volume of 200 µl. Metabolic oxidation was measured by alamarBlue reduction on a fluorescent plate reader (Biotek FLX-800) at excitation of 540 nm and emission of 600 nm; fluorescence was read at 30 min intervals at 37°C with continuous shaking for 14 h. Bacteria remaining in the presence of neutrophils were determined by comparing bacterial growth to that of a range of bacteria concentrations without neutrophils and fit to a log-linear standard curve based on the time to half-maximal growth.

### Neutrophil functional and chemotaxis assays

Cells were resuspended in neutrophil medium (RPMI containing 2% HIPPP, and 10 mM HEPES, pH 7.6) or in Krebs-Ringer phosphate-buffered saline with dextrose (KRPD; 154 mM NaCl, 5.6 mM KCl, 1.1 mM MgSO_4_, 2.2 mM CaCl, 0.85 mM NaH_2_PO_4_, 2.15 mM Na_2_HPO_4_ and 0.2% dextrose) for individual functional assays. A cytochrome c reduction assay for superoxide anion (O_2_^−^) release[31] was employed in neutrophils stimulated with a 1∶1 m.o.i. as indicated. Dual phosphorylation of p38 MAPk was determined by ELISA (ELISATech) [32] from 4.8×10^6^ neutrophils stimulated with each bacterial species (1∶1 ratio) for a range of times from 0 to 2 h. Neutrophil necrosis was determined by the percent of total lactate dehydrogenase (LDH) released (Cytotoxicity Detection Kit; Roche). Apoptosis was quantified by immunoassay for cytoplasmic histone-associated DNA fragments (Roche), and reported as an apoptosis index, the ratio of histone-bound DNA in a given sample to the level of histone-bound DNA detected in an equivalent number of cycloheximide-stimulated circulatory neutrophils, which were >95% morphologically apoptotic. Standard ELISA assays (ELISATech) were used to quantify release of cytokines. Measurements of O2^−^ release were taken following 1 h, interleukin-8 (IL8) release following 2 h, TNF release and cell death from apoptosis or necrosis were measured at 4 h. Migration assays were performed on calcein-labelled neutrophils for 1 h as described previously [32] and reported as percent of total neutrophils migrating through the membrane. Area-under-the-curve (AUC) was calculated for each migration curve as an index of overall migration.

### Data analysis

Data were analyzed using GraphPad Prism software. The significance of the difference was assessed by non-parametric Mann-Whitney U test for comparisons between normal ISG expression and high ISG expression. To accurately depict the extent of neutrophil variability, data is presented as individual data points and median for the normal and high ISG groups. For all tests, *p*<0.05 was considered significant.

## Results

### Anti-viral gene expression in circulating neutrophils isolated from ARDS patients

**Figure 1:**
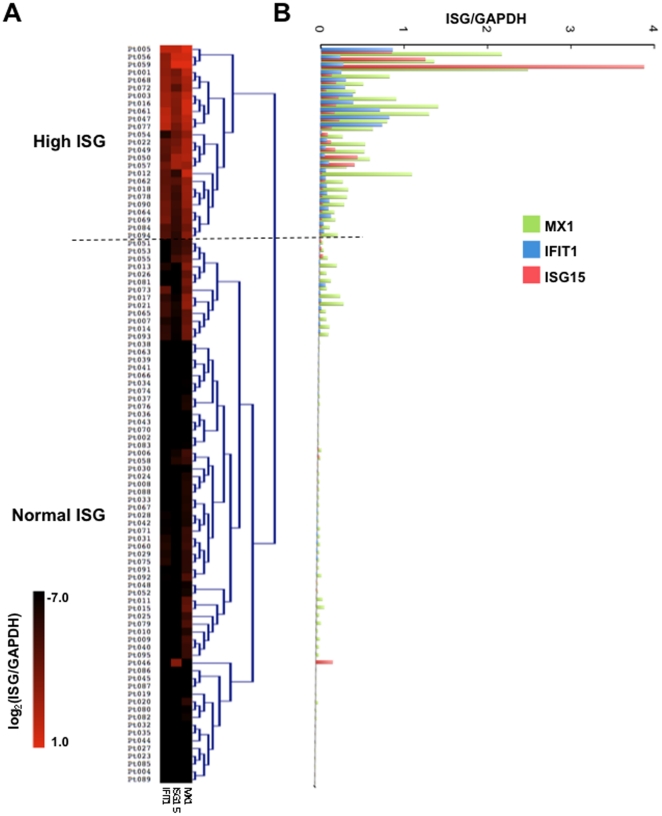
ARDS patients include a cohort of individuals with elevated neutrophil ISG expression. Expression of neutrophil *MX1*, *IFIT1*, and *ISG15* were determined by real-time PCR and normalized to expression of *GAPDH*. Log_2_-transformed, normalized ISG levels were ordered by hierarchical clustering, with the primary clusters designated as either high or normal ISG expression. The dotted line indicates the separation of the normal and high ISG groups. Individual ARDS patients (Pt) are indicated. The difference between the cohorts was highly significant as indicated in the text. (**A**) The heat-map indicates the range of log2-transformed data. (**B**) Non-transformed data is aligned to the clustering results for an alternative visualization.

Using three prototypical ISGs (*MX1*, *ISG15*, and *IFIT1*) as markers of overall ISG expression [33], relative expression levels were determined by quantitative PCR. These three genes are a feasible and representative sample of ISGs induced by Type I interferons and viral infections, and are among the three most highly expressed ISGs in human neutrophils [34], [35] and other leukocytes [23]–[25], [33]. ARDS patients demonstrate variable expression of *MX1*, *ISG15*, and *IFIT1* (**Figure 1**). In order to test phenotypic characteristics associated with high ISG expression, unsupervised hierarchical cluster analysis was performed to classify these subjects. Hierarchical cluster analysis represents an unbiased method to distinguish groups based on multiple variables. Expression of each gene normalized to *GAPDH* expression was log2 transformed and sorted by Euclidean distance (see Methods). Two major clusters were delineated on the basis of this analysis, termed “normal” and “high” ISG expression. The high ISG cluster contained 25 of 95 subjects (26%) (**Figure 1**). An independent method of grouping, *k*-means clustering, returned the identical high ISG group (not shown). Expression values of each of the three genes was significantly greater in the high group than the normal group (MX1: 0.036±0.055 vs 0.719±0.553, p = 9.4×10^−15^; ISG15: 0.007±0.024 vs 0.353±0.788, p = 0.0003; IFIT1: 0.006±0.011 vs 0.255±0.259, p = 2.1×10^−12^; values are means ± SD of the ISG/GAPDH ratio for normal vs high expressing groups).

Demographic and clinical features of patients assigned to the normal and high ISG cohorts were compared (**Table 1**). The age, gender distribution, and proportion of ARDS patients presenting with primary sepsis or pneumonia were not different (**Table 1**). As expected, a significantly greater proportion of subjects in the high ISG cohort were determined to have viral infections. However, there was no difference in incidence of pre-existing conditions associated with elevated ISG expression, including chronic dialysis, immunosupression, and diabetes (**Table 1**).

**Table 1:** Study Population.

Cohort	(n)	Age (yrs)	Males (%)	Viral Infection^1^ (%)	Sepsis or pneumonia (%)	Chronic dialysis (%)	Immuno-compromised (%)	Diabetes (%)
**ARDS Patients** Median (range)	95	52.0 (21–86)	52.6	15.8	77.9	4.2	4.2	28.4
***Normal ISG*** Median (range)	70	52.5 (21–82)	54.3	8.6	75.7	4.3	1.4	28.6
***High ISG*** Median (range)	25	50.0^NS^ (21–86)	48.0^NS^	36.0^2^	84.0^NS^	4.0^NS^	12.0^NS^	28.0^NS^

1 Viral Infection: Confirmed Influenza A, HIV, acute HSV, or Hepatitis C.

NS Not Significantly different than subjects in the normal ISG cohort.

2 *p* = 0.003 Fisher's Exact Test (2-Tail).

### ISG expression is associated with circulating neutrophil dysfunction in ARDS

Circulating neutrophils responses previously implicated in the pathogenesis of ARDS were examined following exposure to live *S. aureus,* a gram-positive bacterium frequently identified in post-viral infections, and to *P. aeruginosa*, an opportunistic Gram-negative bacterium that causes secondary nosocomial infections in critically ill patients. A broad functional phenotype of circulating neutrophil response was tested in the context of normal and high ISG expression.

#### IL8-induced Migration

**Figure 2:**
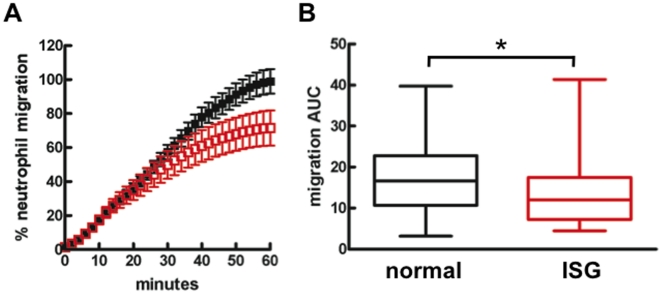
High ISG expression is associated with reduced circulating neutrophil migration in ARDS patients. (**A**) Directional migration towards IL8 was measured in isolated neutrophils with a modified Boyden chamber system over 60 minutes and (**B**) AUC of migration curves shown in (A); ARDS patients with high ISG expression (ISG; *red*; n = 20) were found to have reduced migration compared to those with normal ISG expression (*black*; n = 58). Bar and whiskers indicate median, 25^th^- and 75^th^-percentile, and range. *, p<0.05.

Circulating neutrophils isolated from patients with ARDS or sepsis have previously been reported to demonstrate reduced migration [15]. In response to IL8, neutrophils from ARDS patients with high ISG expression demonstrated a reduction in migratory response compared to ARDS neutrophils with normal ISG expression (**Figure 2**).

#### Activation of p38 MAPk

**Figure 3:**
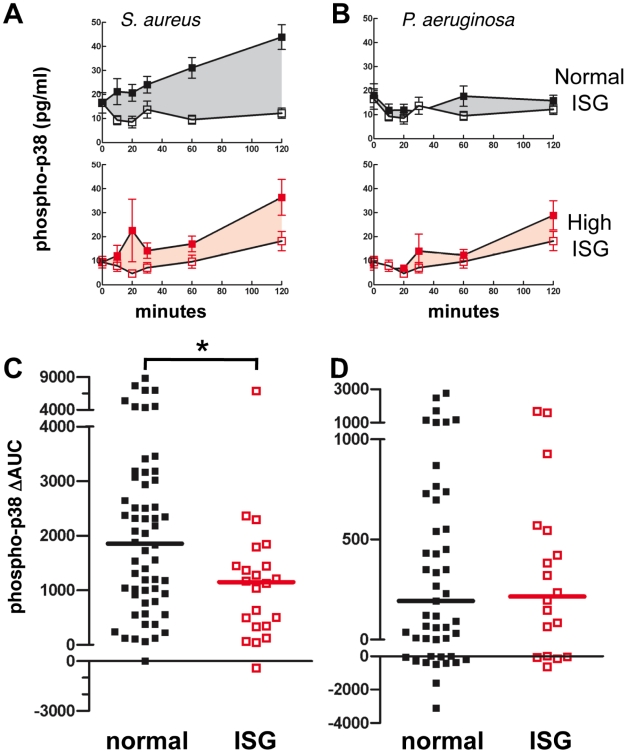
*S. aureus*-induced p38 MAP kinase activity is attenuated in circulating neutrophils with high ISG expression. Time-course of phospho-p38 MAPk phosphorylation induced by (**A**) *S. aureus*, or (**B**) *P. aeruginosa* was measured by ELISA at 6 time points over 2 hours; stimulated *(closed* symbols) or unstimulated neutrophils (*open* symbols) and either normal (*black*; *upper* panels) or high ISG expression (*red*; *lower* panels). The shaded portion indicates the ΔAUC. Mean ± SEM is shown. Phospho-p38 MAPk ΔAUC values from a phospho-p38 MAPk time-course were determined in neutrophils in response to (**C**) *S. aureus*, and (**D**) *P. aeruginosa* with normal (*black*) and high ISG expression (*red*). Median values are indicated by the like-colored bars; ARDS normal ISG (n = 58 in panels A and C, and 49 in panels B and D), ARDS high ISG (n = 22 in panels A and C, and 18 in panels B and D). *, p<0.05.

As a central intracellular signaling mechanism of neutrophil stress responses, p38 MAPk regulates O_2_^−^ release, apoptosis, migration, and cytokine production to inflammatory stimuli [36], [37]. Dual phosphorylation of p38 MAPk correlates with kinase activity. Bacterial-induced p38 MAPk activation was quantified as an area under the curve (AUC) of stimulated phosphorylation over a 2-hour exposure to the bacteria (**Figure 3**). In response to *S. aureus*, neutrophils isolated from ARDS patients with high ISG expression had significantly attenuated p38 MAPk activation compared to those with normal ISG expression (**Figure 3C**). Unstimulated p38 MAPk activation was not different between high and normal ISG expression (**Figure 3A,B**). Compared to *S. aureus,* exposure to *P. aeruginosa* induced less p38 MAPk activation under the conditions studied, and no difference was seen between normal and high ISG-expressing groups (**Figure 3D**).

#### Superoxide anion release

**Figure 4:**
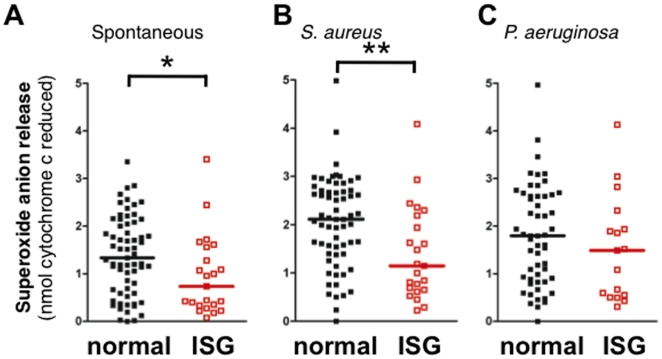
Superoxide anion release is suppressed in neutrophils expressing ISG. Release of superoxide anion (O_2_^−•^) from neutrophils following 60 minutes incubation. (**A**) Spontaneous; (**B**) *S. aureus*-induced; and (**C**) *P. aeruginosa*-induced release of O_2_^−•^. ARDS patients demonstrated significant attenuation of O_2_^−•^ release when ISG expression was elevated in non-stimulated and *S. aureus*-stimulated neutrophils. Scatter plot of values in neutrophils with normal (*black*) and high ISG expression (ISG; *red*); normal ISG (n = 65 in panels A and B, and 55 in panel C), high ISG (n = 23 in panels A and B, and 17 in panel C). *p<0.05, **, p<0.01.

The release of superoxide anion (O_2_^−^) by circulating neutrophils from ARDS patients was tested in response to *S. aureus* and *P. aeruginosa* (**Figure 4**). Neutrophils isolated from ARDS patients with high ISG expression spontaneously produced significantly less O_2_^−^ than normal ISG expressing cells (**Figure 4A**) and in response to *S. aureus* (**Figure 4B**). O_2_^−^ release in response to *P. aeruginosa* was not different between groups of neutrophils (**Figure 4C**). Phorbal myristate acetate, a potent activator of reactive oxygen intermediate production, induced similar levels of O_2_^−^ release in both groups (data not shown), suggesting that the potential to release O_2_^−^ has remained intact, independent of ISG expression.

#### Cytokine production

**Figure 5:**
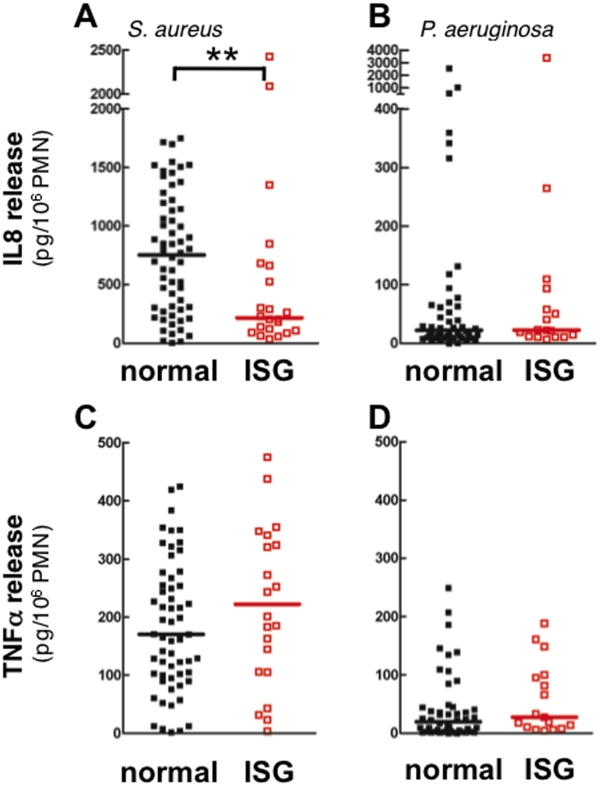
S. aureus-induced IL8 release is reduced in high ISG-expressing neutrophils from ARDS patients. Release of IL8 following 2 h incubation with (**A**) *S. aureus*, and (**B**) *P. aeruginosa*. TNF release following 4 h incubation with( **C**) *S. aureus,* and (**D**)* P. aeruginosa*. Cytokine release from neutrophils with normal (*black*) and high ISG expression (ISG; *red*) are shown; normal ISG (n = 52 in panels A and C and 42 in panels B and D), high ISG (n = 19 in panels A and C and 14 in panels B and D); *p<0.05.

Neutrophil release of IL8 and TNF was tested following exposure to *S. aureus* and *P. aeruginosa*. Both bacteria stimulated significant release of IL8 and TNF over non-stimulated neutrophils (data not shown). Reduced secretion of IL8 was observed by circulating neutrophils isolated from ARDS patients with high ISG expression in response to *S. aureus* (**Figure 5A**), but no difference in IL8 release was observed in response to *P. aeruginosa* (**Figure 5B**). In contrast, high ISG-expression was not associated with alterations in TNF release following exposure to *S. aureus* or *P. aeruginosa* (**Figure 5C,D**), indicating that the ability to secrete cytokines is not universally impaired in ISG-expressing neutrophils.

#### Neutrophil death

**Figure 6:**
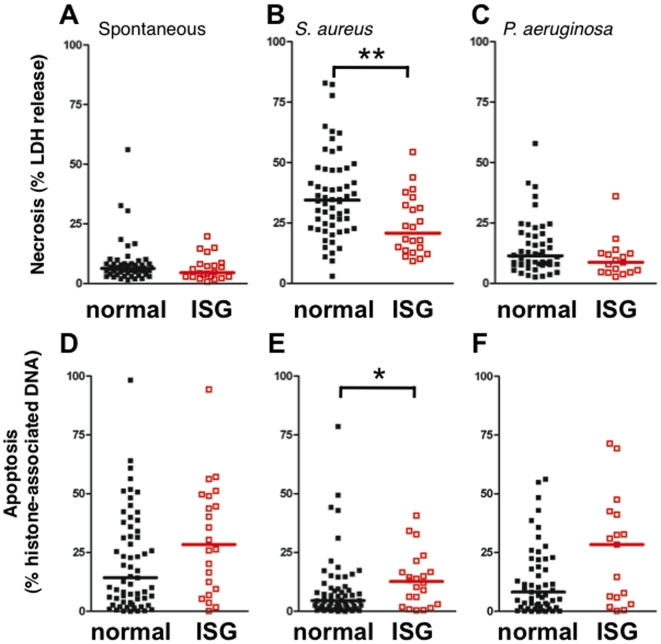
Reciprocal modification of cell death in neutrophils from ARDS patients expressing ISG. (**A–C**) The release of LDH to assess necrosis, and (**D–F**) percent of soluble histone-associated DNA fragments to assess apoptosis, from isolated neutrophils after 4 h treatment. (**A**) Spontaneous necrosis. (**B**) *S. aureus*-induced necrosis. (**C**) *P. aeruginosa*-induced necrosis. (**D**) Spontaneous apoptotic cell death (**E**) *S. aureus*-induced apoptotic cell death. (**F**) *P. aeruginosa*-induced apoptotic cell death. Scatter plot of values in neutrophils with normal (*black*) and high ISG expression (ISG; *red*); median values are indicated by the like-colored bars; normal ISG (n = 50–63), high ISG (n = 17–22). *, p<0.05, **, p<0.01.

Pathways leading to cell death have a complex effect on inflammation and pathogen killing by the short-lived neutrophil. While necrotic death is implicated in airway injury, apoptotic neutrophil accumulation may dampen the inflammatory response [38]. In addition, highly virulent strains of *S. aureus* have been identified which evoke neutrophil lysis following phagocytosis [39], [40]. Circulating neutrophils from ARDS patients with high ISG expression had significantly less necrotic cell death following 4 h exposure to *S. aureus* (**Figure 6B**), but necrotic cell death was not changed in response to *P. aeruginosa* (**Figure 6C**), or when unstimulated (**Figure 6A**).

An opposite pattern was observed in analysis of apoptosis. Circulating neutrophils from ARDS patients with high ISG expression demonstrated greater apoptotic cell death following 4 h exposure to *S. aureus* (**Figure 6E**); however, no change was observed in unstimulated cells or following exposure to *P. aeruginosa* (**Figure 6D,F**). Together, Figure 6 demonstrates that high ISG expression in neutrophils treated with *S. aureus* from ARDS patients has an overall effect of enhancing apoptosis and reducing necrosis. Furthermore, an interaction between type of cell death and ISG expression was evident for *S. aureus*- (p<0.01) and *P. aeruginosa*-treated (p<0.05) neutrophils when tested by two-way ANOVA. Total cell death, as assessed by adding the percentage of cells undergoing necrosis and apoptosis, was not different for any condition (data not shown).

#### Bacterial killing

**Figure 7:**
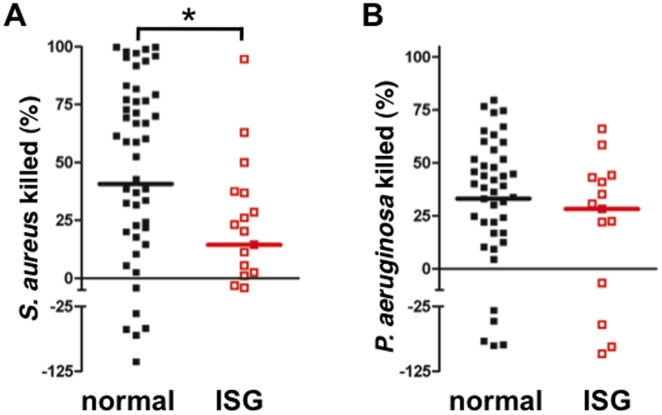
High ISG expression is associated with impaired neutrophil killing of *S. aureus* in ARDS. Killing of (**A**) *S. aureus* and (**B**) *P. aeruginosa*. Bacteria were exposed to adherent neutrophils for 60 min, and bacteria remaining was compared to that in the absence of neutrophils to determine percentage of bacteria killed. Values less than zero indicate growth of bacteria during the assay. ISG expression was associated with an impaired bactericidal activity against *S. aureus*, but not against *P. aeruginosa*. Scatter plot of values in neutrophils with normal (*black*) and high ISG expression (ISG; *red*); median values are indicated by the like-colored bars; normal ISG (n = 54 in panel A, and 49 in panel B), ARDS high ISG (n = 19 in panel A, and 15 in panel B); *, p<0.05.

Reduced p38 MAPk activation and O2^−^ release, combined with enhanced apoptosis, suggests that high ISG expression may be associated with reduced bactericidal capacity of these neutrophils. We assayed survival of bacteria in the presence of neutrophils to determine bactericidal activity, which includes phagocytic and non-phagocytic killing pathways. Circulating neutrophils from ARDS patients that expressed normal ISG levels killed *S. aureus* efficiently, while high ISG expression was associated with impaired bactericidal ability (**Figure 7A**). Killing of *P. aeruginosa* was not reduced in high-ISG expressing neutrophils (**Figure 7B**).

## Discussion

This study is the first known exploration of the role of neutrophil ISG expression in ARDS. ISGs were first identified as a consequence of Type I interferon activation of cells. Detection of virus or viral products leads to IFNα/β release, but may occur also in response to bacterial infections and autoimmune diseases [41]. Subsequent induction of ISGs conveys the anti-viral capacity of IFNα/β by producing gene products affecting viral replication and release, and inflammatory and immunomodulatory cytokines [20], [21]; however, this diverse gene family likely has broader immunomodulatory effects [42]–[45]. To determine if ISG expression alters neutrophil function in ARDS patients we interrogated a three-gene panel of ISGs, previously shown to be highly expressed in stimulated neutrophils [34], [35], [46], lymphocytes [23], peripheral blood mononuclear cells [24], [25], and whole blood [33]. Our analysis indicates that approximately three-quarters of ARDS patients have negligible upregulation of ISG, while a quarter have robust ISG expression. Unsupervised hierarchical clustering of *MX1*, *ISG15*, and *IFIT1* expression objectively defined in an unbiased manner a distinct high ISG-expressing group. A second, independent clustering approach returned an identical group.

We hypothesized that the immunomodulatory effect associated with elevated ISG expression may extend to circulating neutrophil responses in ARDS. Neutrophils from ARDS patients were analyzed for a series of functional responses to live bacteria that may be clinically and mechanistically relevant to the syndrome. Interestingly, neutrophils from ARDS patients with elevated ISGs were less adept at killing a Gram-positive bacterium, *S. aureus*, but not a Gram-negative bacterium, *P. aeruginosa*. A mechanistic basis for impaired bacterial killing may in part be linked to attenuation of O2^−^ generation, which is especially important for successful eradication of *S. aureus* [47]. Our killing assay measures the summation of all possible killing mechanisms, and further study is needed to assign a specific mechanism to this effect. Furthermore, high ISG expression was found to shift neutrophils towards apoptotic cell death and away from necrotic cell death (Figure 6). Intriguingly, highly virulent strains of *S. aureus* are capable of evoking significant post-phagocytic necrosis over time periods longer than what was studied in this report [39], [40]. Apoptotic cells are associated with anti-inflammatory potential [38], and therefore may restrain beneficial effects of other cells. IFNα/β have been shown to sensitize a number of cell types to apoptosis following bacterial infection, as seen in *Listeria* infected murine macrophages [48]. Thus, our data showing enhanced apoptosis following bacterial infection of ISG-expressing neutrophils extends this phenomenon to human cells. Furthermore, neutrophil extracellular trap (NET)-mediated cell death was not addressed, although IFNα acutely sensitizes neutrophils to NET formation [49].

Further evidence of an impaired circulating neutrophil phenotype associated with high ISG expression comes from studies of migration, cytokine release, and p38 MAP kinase activation. Neutrophils from ARDS patients have reduced migration compared to healthy controls [15](data not shown), and migration was further worsened in cells with high ISG expression. In addition, IL8 secretion from *S. aureus*-treated neutrophils was selectively reduced in the high ISG group, while TNF release was not altered, supporting the conclusion that the decrease in IL8 release was not a nonspecific effect of reduced neutrophil viability. As with O2^−^ release, neutrophil migration and release of IL8 are dependent on p38 MAPk activation [37]. Accordingly, we detected suppression of p38 MAPk activation by *S. aureus* in neutrophils with high ISG expression.

The more pronounced effect of high ISG expression of ARDS neutrophils in response to *S. aureus* than *P. aeruginosa* may form a framework for a difference in susceptibility to various post-influenza infections. Clinical data and animal models demonstrate that viral infections increase susceptibility to secondary bacterial infections [26], [50], [51], even in the presence of neutrophil-rich inflammation. The classic scenario of post-viral *S. aureus* pneumonia is familiar to clinicians [26], whereas *P. aeruginosa* has not been implicated in this setting.

Neutrophils are known to respond to IFNα with STAT phosphorylation and upregulation of ISGs [28], [35], [46], [52]. Interestingly, neutrophils are a major contributor of ISG expression in LPS-exposed whole murine lung [53]. Wright *et al*. [35] reported the reversible induction of ISGs in neutrophils during periodontitis associated with enhanced reactive oxygen intermediate production. Other investigators have demonstrated inhibition of apoptosis and enhanced reactive oxygen intermediate production in neutrophils treated acutely with IFNα/β [52], [54]–[56]. Although the exact mechanism is unknown, neutrophil dysfunction may derive from the activities of the diverse ISG products that establish an anti-viral phenotype, which possibly have additional effects in combination with the potent inflammatory environment of ARDS.

Although we have no data to support a causal role of ISG expression in neutrophil dysfunction in ARDS, several studies suggest that IFNα/β signals and ISGs promote anti-bacterial activity. Difficulties in testing this hypothesis in neutrophils include reproducing the developmental stage and the already highly activated state of ARDS neutrophils, and in genetically altering neutrophils. However, ubiquitin-like ISG15 modifies proteins [57], including signaling proteins, and the ability of secretory murine ISG15 to act as a neutrophil chemoattractant suggests an inflammatory role [58]. Anti-bacterial mechanisms are not defined for MX1, which codes for a dynamin-like GTPase that may interfere with viral assembly [59], while IFIT1 inhibits translation by interacting with eukaryotic initiation factor 3 [60]. These particular ISGs may play a part an anti-bacterial role, or be upregulated in coordination with other ISGs directly responsible for the effect. Alternatively, neutrophil dysfunction may be mechanistically independent of ISGs. The ability of Type I interferons to regulate anti-bacterial activity is beginning to be appreciated. *Ifnb* ^−^/^−^ and *Ifnar1* ^−^/^−^ mice are more susceptible to *Streptococcal* infection, although *Ifnar1* ^−^/^−^ mice are protected from infection by *Listeria*, *Chlamydia* and in polymicrobial sepsis [42]–[45]. Thus, immunomodulation by IFNα/β may depend on the specific infection. We are unaware of studies determining the role of Type I interferons on neutrophils or in human disease in response to *S. aureus* or *P. aeruginosa*, but our data suggest that IFNα/β signaling results in an impairment of neutrophil bactericidal activity specifically to *S. aureus*. Likewise, the dysfunctional properties of ISG-expressing neutrophils in response to live bacteria may predict similar dysfunction in response to other inflammatory factors, which could contribute to the acute organ failure prominent in ARDS.

In the majority of patients the source of ISG induction has not been identified. These initial experiments were designed to determine only the effect of ISG expression with neutrophil functional responses. In the course of clinical care, a significantly greater fraction of subjects were identified as having serious vial infections in the high ISG cohort (**Table 1**). But high ISG expression was not associated with sepsis or pneumonia **(** **Table 1** **)**. Future studies are warranted to systematically screen for viral infections in this population. Recently, Limaye et al. [61] observed cytomegalovirus reactivation in critically ill patients, although notably, detection occurred one to two weeks after hospitalization, while our samples were collected within 72 h.

The functional response of neutrophils to any chosen stimulus is remarkably variable. Differences in neutrophil functional responses [9], [12], [14], [15], [17] and in mechanistically relevant molecules [10], [11] have been described previously in ARDS patients as a whole, yet variability in presentation, severity, and response to therapy remain poorly understood. The data presented here suggest that ISG expression could underlie, in part, variability in ARDS. The battery of cytokines, damage-associated molecular patterns, and other pro-inflammatory insults intrinsic to ARDS may modify the response phenotype mediated by ISG upregulation. Therefore, high ISG expression may represent a “first hit” that, in combination with the ARDS pro-inflammatory milieu, diminishes neutrophil responses. Thus, our data suggest that the altered-response phenotype associated with elevated ISG expression should be considered along with genetic, environmental, and complex demographic factors as a contributor to heterogeneity of neutrophil response in ARDS. Further studies targeting the Type I interferon pathway may identify specific molecular mechanisms of neutrophil dysfunction in ARDS. Studies are currently underway to analyze neutrophil ISG expression as a marker of ARDS outcome, and the role of high ISG expression in other neutrophil-mediated diseases.
